# Full GMP-Compliant Validation of Bone Marrow-Derived Human CD133^**+**^ Cells as Advanced Therapy Medicinal Product for Refractory Ischemic Cardiomyopathy

**DOI:** 10.1155/2015/473159

**Published:** 2015-10-01

**Authors:** Daniela Belotti, Giuseppe Gaipa, Beatrice Bassetti, Benedetta Cabiati, Gabriella Spaltro, Ettore Biagi, Matteo Parma, Andrea Biondi, Laura Cavallotti, Elisa Gambini, Giulio Pompilio

**Affiliations:** ^1^Laboratory of Cell Therapy “Stefano Verri”, Azienda Ospedaliera San Gerardo, Via Pergolesi 33, 20900 Monza, Italy; ^2^Department of Health Science, Università degli Studi di Milano-Bicocca, piazza dell'Ateneo Nuovo 1, 20126 Milan, Italy; ^3^Tettamanti Research Center, Azienda Ospedaliera San Gerardo, Via Pergolesi 33, 20900 Monza, Italy; ^4^Laboratory of Vascular Biology and Regenerative Medicine, Centro Cardiologico Monzino, IRCCS, Via Parea 4, 20138 Milan, Italy; ^5^Department of Pediatrics, MBBM Foundation, Ospedale San Gerardo, Via Pergolesi 33, 20900 Monza, Italy; ^6^Department of Ematology, Azienda Ospedaliera San Gerardo, Via Pergolesi 33, 20900 Monza, Italy; ^7^Department of Cardiovascular Surgery, Centro Cardiologico Monzino, IRCCS, Via Parea 4, 20138 Milan, Italy; ^8^Department of Clinical Sciences and Community Health, Università degli Studi di Milano, Via Festa del Perdono 7, 20122 Milan, Italy

## Abstract

According to the European Medicine Agency (EMA) regulatory frameworks, Advanced Therapy Medicinal Products (ATMP) represent a new category of drugs in which the active ingredient consists of cells, genes, or tissues. ATMP-CD133 has been widely investigated in controlled clinical trials for cardiovascular diseases, making CD133^+^ cells one of the most well characterized cell-derived drugs in this field. To ensure high quality and safety standards for clinical use, the manufacturing process must be accomplished in certified facilities following standard operative procedures (SOPs). In the present work, we report the fully compliant GMP-grade production of ATMP-CD133 which aims to address the treatment of chronic refractory ischemic heart failure. Starting from bone marrow (BM), ATMP-CD133 manufacturing output yielded a median of 6.66 × 10^6^ of CD133^+^ cells (range 2.85 × 10^6^–30.84 × 10^6^), with a viability ranged between 96,03% and 99,97% (median 99,87%) and a median purity of CD133^+^ cells of 90,60% (range 81,40%–96,20%). Based on these results we defined our final release criteria for ATMP-CD133: purity ≥ 70%, viability ≥ 80%, cellularity between 1 and 12 × 10^6^ cells, sterile, and endotoxin-free. The abovementioned criteria are currently applied in our Phase I clinical trial (RECARDIO Trial).

## 1. Introduction

In the last decade, bone marrow (BM) and peripheral blood- (PB-) derived CD133^+^ endothelial progenitor cells have been tested in controlled clinical trials as therapeutic agent for heart failure both in acute [1, 2] and chronic [3, 4] setting, with the aim to achieve neoangiogenesis in ischemic myocardial territories. Published Phase I and Phase II studies, although heterogeneous in terms of revascularization strategies and cell delivery routes, reported a partial or complete restoration of global left ventricular function (LV) and improvements in regional myocardial perfusion [1, 5–13]. A number of adequately powered controlled Phase II and Phase III trials are currently ongoing to confirm these preliminary clinical evidences [14].

Mechanistically, a large body of preclinical evidence has shown that CD133^+^ cells, a subset of CD34^+^ progenitors [15, 16], exert their mode of action in ischemic tissues by directly differentiating into newly forming vessels [17] and, predominantly, by indirectly activating proangiogenic signaling through indirect paracrine mechanisms [7, 18]. Due to their nonhomologous use, notwithstanding immunomagnetically clinical-grade purified [19], CD133^+^ progenitors [20] have to be considered in the cardiovascular setting as an Advanced Therapy Medicinal Products (ATMP), in compliance with the European Medicine Agency (EMA) guidelines [21] and the Committee of Advance Therapies (CAT) Reflection paper on human stem cell-based medicinal product [22]. As ATMP, CD133^+^ cells require manipulation in certified facilities operating with pharmaceutical standards in order to ensure high-quality and safety manufacturing processes in compliance with Good Manufacturing Practice (GMP) criteria [23]. Specifically, the final CD133^+^ cell product must be released upon a strict manufacturing characterization, as well as definition of release criteria and quality controls. This validation process is in fact the prerequisite for the release of batches intended for clinical use.

Importantly, in the cardiac cell therapy field the ATMP validation process relies upon intrinsic features of the starting material. It is in fact well known how the number of BM progenitors, their viability, and functionality may be severely affected by multiple cardiovascular risk factors of self-donor patients with ischemic heart failure [24, 25]. In a previous proof-of-concept paper, we have reported that a GMP-compliant implementation of cord-blood- (CB-) derived CD133^+^ cells for cardiovascular repair does not alter the angiogenic potency* in vitro* and* in vivo* [26]. Using BM of patients with ischemic cardiomyopathy as starting material, we have here developed a standardized final GMP-compliant clinical-grade manufacturing protocol for human CD133^+^ cells fulfilling clinical-grade ATMP standards (ATMP-CD133). Data generated in the present work have been included in the Quality Section of the Investigational Medicinal Product Dossier (IMPD) “ATMP-CD133” [21], recently cleared by the competent Italian Authority (Istituto Superiore di Sanità, Rome, Italy) as therapeutic agent of the actively enrolling Phase I clinical trial RECARDIO trial [27].

## 2. Materials and Methods

### 2.1. Quality Documentation Concerning ATMP-CD133

IMPD quality documentation structure has been set up following EMA guidelines (CHMP/QWP/185401/2004). Specifically, our active ingredient consists of human BM-derived CD133^+^ endothelial progenitor cells (drug substance). The drug substance resuspended in physiological saline plus 5% of human serum albumin (HSA) represents the ready-to-use ATMP medicinal product (ATMP-CD133).

### 2.2. Equipment and Facility Characteristics

Manufacturing and quality control test have been performed in a GMP cell production facility (Laboratory of Cell Therapy “Stefano Verri”, Monza, Italy) authorized by the Italian Competent Authority (Agenzia Italiana del Farmaco, AIFA) with the licence aAMM-70/2013, according to European and Italian regulatory rules [23]. In particular, all cell manipulations were assessed in B/A GMP classes.

### 2.3. Assessment of Risk Analysis

An* ad hoc* risk analysis was assessed according to international guidelines [28], with particular focus on the Investigational Medicinal Products (IMP) [29]. Unsuitable starting material, unmet quality standards of starting raw materials, manufacturing process contamination and cross-contamination, positive selection failure, overnight storage and transportation of ATMP-CD133, and miss-labelling were identified as in-process critical steps. Strategies to specifically avoid and manage those steps have been developed and documented in IMPD Standard Operative Procedures (SOPs). As an example, a worst case scenario of production process failure has been considered. This procedure allows to recover a discrete number of CD133^+^ cells found in the negative fraction, as the negative fraction bag is stored at 4°C until batch release, and a supplemental back-up CliniMACS kit is available at the Cell Factory for emergencies. An evaluation of adventitious risk contamination was also performed [30].

### 2.4. Aseptic Validation Media Fill

The aseptic process has been validated according to the European GMP legislation [29]. Media fill validation is required in order to ensure robustness, reproducibility, and safety of manufacturing process. In the simulation (i.e., media fill), all aseptic operations were performed using Tryptic Soy Broth (TSB) according to SOPs. At the end of the simulation all collected samples were tested for sterility according to Eu.Ph.2.6.1. To verify the potential microbial growth, samples collected were incubated at +20–25°C for 7 days followed by 7 days at +30–35°C. During all this time (14 days) culture medium turbidity was observed. Subsequently, growth promotion was performed on media pool to verify the fertility of the medium and the absence of growth inhibition factors, according to Eu.Ph.2.6.1.

### 2.5. Premises and Environment Condition during Manufacturing Process

Premises were fully compliant with GMP rules in order to protect the manufacturer and to minimize risk of cross-contamination [23]. Working conditions of premises such as temperature, humidity, and pressure were maintained and controlled by DESIGO system (Siemens, USA). Adjacent rooms of different grades have been maintained with a controlled differential pressure of 10–15 Pascal according to EU guidelines for GMP [23]. Manufacturing process was set up in A and B classes (classified according to EN ISO 14644-1), and maximum permitted airborne particle concentration was also monitored taking into account limitations as indicated above [29]. Aseptic conditions were monitored using methods such as settle plates, volumetric air samples, and surface sampling [29].

### 2.6. Collection and Storage of the BM Cells

From May 2009 to July 2011, 8 patients suffering for end-stage refractory myocardial ischemia have been authorized by our local Ethic Committee to receive as compassionate therapy direct intramyocardial injections of ATMP-CD133 according to Italian national laws. The collection of BM was performed after obtaining written informed consent from the patient. Patients' clinical profile is depicted in Supplemental Table 1 in Supplementary Material available online at http://dx.doi.org/10.1155/2015/473159. Bone-marrow blood has been collected from patient's posterior iliac crest in a sterile fashion. Filled syringes were then transferred in a bag containing heparin (final concentration ≥ 5 U/mL). Needles were finally removed by a direct pressure around the collection area. Bag containing fresh bone marrow sample has been weighted, labeled, and shipped to the cell factory. Bag's shipping was performed at controlled and recorded temperature (+4°C/+20°C) using a rigid box according to defined GMP SOPs. The time limit for bag delivery has been set at 36 hours from collection. Production process was started immediately after material reception without storage.

### 2.7. ATMP-CD133 Manufacturing Process

**Figure 1:**
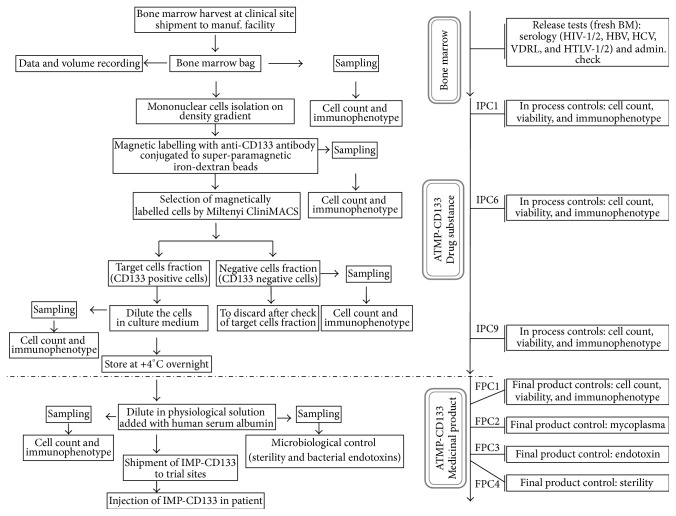
Manufacturing process from the drug substance to the medicinal product. The figure summarizes using a flowchart the entire process from the collection of the patient's bone marrow to the injection of the medicinal product back to the patient.

The production of ATMP-CD133 was performed the day before injections in a semiclosed system (CliniMACS Miltenyi Biotech, GmbH, Germany) according to manufacturer's instructions. Briefly, mononucleated cells (MNCs) were isolated from BM by density gradient centrifugation using Ficoll-Paque Plus (GE Healthcare Life Sciences, UK). After washing with phosphate-buffer saline containing 0.5% (v/v) HSA (PBS-HSA) and centrifugation at 600 ×g for 15 minutes, cells were resuspended into 25 mL volume with PBS-HSA containing 3.5 mL of anti-CD133 antibody (Miltenyi Biotec, GmbH, Germany) conjugated with dextran-magnetic microbeads and 1.5 mL of human IgG. Cells were incubated for 30 min at room temperature under gentle agitation. After washing (600 ×g, 15 minutes), cells were resuspended in 100 mL of PBS-HSA, counted, and characterized for immunophenotype and CD133^+^ endothelial progenitors were selected using the CliniMACS Magnetic Separation device (Miltenyi Biotec, GmbH, Germany). Both CD133^+^ and CD133^−^ fractions were finally collected in separated bags. Each fraction undergone quality control checks such as cell count, immunophenotype, and viability. Magnetically selected CD133^+^ cells were then resuspended in X-VIVO15 (Lonza, Switzerland) and stored at +4°C overnight in a 50 mL polypropylene tube (drug substance). The day after, release tests such as cell count, immunophenotype, and viability were repeated before lot releasing. In addition, sampling for endotoxin and sterility tests were performed. Moreover, an aliquot of CD133^+^ cells was stored in liquid nitrogen for retesting in case of required investigation and/or for research purposes. Remaining cells were washed (600 ×g, 10 minutes) to eliminate X-VIVO15 and resuspended in 10 mL physiological saline plus 5% of HSA. The ready-to-use final product ATMP-CD133 was then shipped, under controlled conditions according to SOPs, to the destination site for clinical use. An outline of manufacturing process as well as relevant quality control steps is reported in Figure 1.

### 2.8. Process Quality Control (QC) Tests

Less than thirty days before the collection of BM, patients were checked for the absence of infective agents such as HIV-1/2, HBV (HBsAg, anti-HBc, and HBs), HCV (anti-HCV-Ab), Treponema pallidum (V.D.R.L.), and HTLV-1/2, according to Directive 2006/17/CE. If serology test for viral infection was confirmed to be negative, starting material, defined as fresh BM sample, was collected and sent to the GMP facility where it was checked, approved, and processed. Similarly, all raw materials and reagents, either biological or disposable, were verified for their compliance to be used in the GMP manufacturing process [23]. QC tests were performed throughout the manufacturing steps both for drug substance (in-process controls, IPC) and medicinal product (final-product controls, FPC) in order to ensure continuous monitoring. In addition, two aliquots of ATMP-CD133 have been stored in liquid nitrogen for retesting in case of required investigation or for research purposes. Thus the final amount of cells assigned to the patient is determined based on these procedures.

Details on each IPC and FPC assessed during the whole process, are described in Supplemental Table 2. Specifically, IPC1, IPC6, IPC9, and FPC1 were fixed to test cell count, viability and immunophenotype on BM, preselection fraction, postselection positive fraction, and final product, respectively. Moreover, endotoxin, sterility, and mycoplasma were assessed on final product (FPC2, FPC3, and FPC4).

Cell count was determined by trypan blue dye exclusion method, viability was performed by Propidium Iodide staining, and immunophenotyping was performed by multiparametric flow cytometry as previously described [26]. Briefly, fluorochrome-conjugated monoclonal antibodies were used in order to determine CD133^+^ cells purity. Characteristics and combination of antibodies are reported in Supplemental Table 3. At least 3.5 × 10^5^ cells were incubated for 20 minutes, at +4/+8°C with fluorescent reagents in PBS, then washed by centrifugation, resuspended in PBS, and analyzed using a flow cytometer (FACS Calibur, Becton Dickinson, CA, USA). A hierarchical gating strategy was adopted to determine purity of CD34^+^/CD133^+^ cells as previously reported [26]. Endotoxin detection was performed by Limulus Amebocyte Lysate (LAL) Test (Lonza) according to recommendations of European Pharmacopoeia (E.P.) 2.6.14. Sterility and mycoplasma detection were assessed in outsourcing according to recommendations of E.P.2.6.1 and E.P.2.6.7, respectively. Finally, to characterize the impurities of the final medicinal product, we reviewed in 2 thawed representative samples the complete immunophenotype of the CD133^−^ fraction testing the presence of B (CD19), T (CD3), and NK lymphocytes (CD56), as well as granulocytes (CD15) and myeloid-monocytes cells (CD14).

### 2.9. ATMP-CD133 Release Criteria

Batches were processed and released according to specification described in Gaipa et al. [26]. Briefly, the release criteria for ATMP-CD133 were purity ≥50%, viability ≥70%, cellularity ≥1 × 10^6^, sterile, endotoxin <0.5 EU/mL, and mycoplasma absence. List of release test and specifications are summarized in Supplemental Table 4.

### 2.10. Stability Test

Stability of ATMP-CD133 was assessed both for drug substance and medicinal product. The stability of the drug substance was validated maintaining cells in X-VIVO15 for 14 hours (h) at controlled temperature between +2 and +8°C. On the other hand the stability of the medicinal product was validated at different time points (6 h, 9 h, and 12 h) maintaining cells in physiological saline + 5% HSA. Viability test was evaluated before and after aforementioned storage conditions to confirm the ATMP-CD133 stability.

## 3. Results

### 3.1. Media Fills

Collectively we performed 9 media fill procedures to assess both initial validation (3 batches) and periodic revalidation (6 batches) during the study period (from November 2008 to December 2011). All media fill batches resulted sterile and was compliant for growth promotion analysis. Results demonstrated that both the procedure and personnel involved were able to maintain and guarantee aseptic conditions during all phases of manufacturing process (data not shown).

### 3.2. BM-Derived CD133^+^ Cells Collection, Purification, and Recovery

A total of 8 BM-derived CD133^+^ cell samples were prospectively manufactured from May 2009 to July 2011. Volumes of BM collections ranged between 228 mL and 420 mL (median 278.50 mL). The mean timespan from collection to reception was 59 minutes ± 21.19 (standard deviation, SD). After cell selection with CD133 monoclonal antibody, the median purity of CD133^+^ cells was 90.60% (range 81.40%–96.20%), largely above the acceptance criteria (≥50%), as described in Table 1. For each batch, viability tests have been performed for both starting materials and final product. Final product viability ranged between 96.03% and 99.97% (median 99.87%), exceeding the minimum threshold of 70% established in our previous experience [26] (see Table 1). Absolute number of CD133^+^ cells during manufacturing process was highly variable among samples. The number of CD133^+^ cells in BM samples varied among patients from 15.40 to 180.69 × 10^6^ (median 55.35 × 10^6^). After density gradient separation of mononuclear cells, a median of 64.89% of CD133^+^ cells was recovered (range 38.25%–85.21%). CD133^+^ median recovery after immunomagnetic selection was 30.05% (range 18.37%–56.77%) as referred to prior CliniMACS selection and 20.62% (range 7.69%–34.98%) as referred to BM starting material, respectively. The presence of unwanted cells in the final product (checked in two batches) was very low (less than 2%; data not shown). A detailed summary of absolute number as well as recovery of CD133^+^ cells through each manufacturing phase is described in Table 2 for each batch.

### 3.3. Results of ATMP-CD133 Release Controls

**Figure 2:**
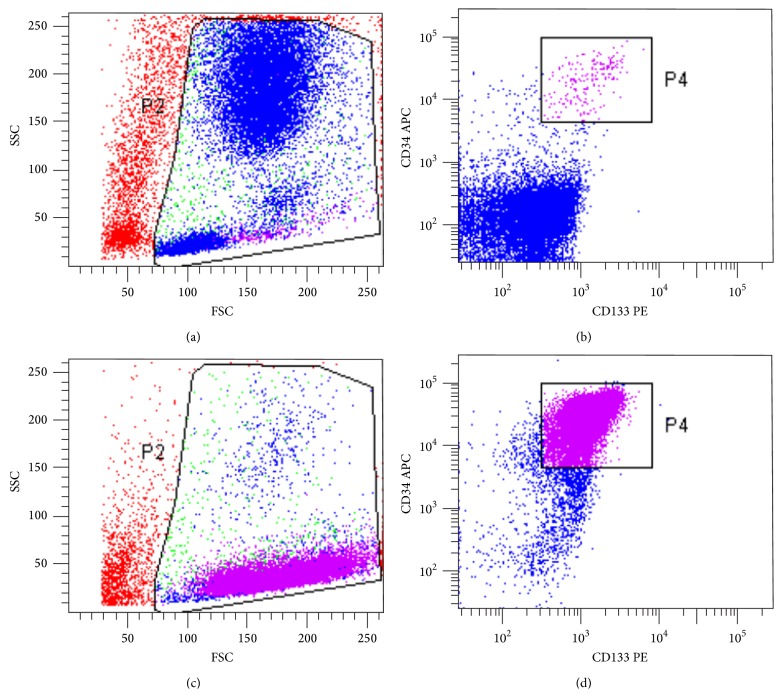
Flow cytometric analysis of CD133^+^ cells. Representative example of purity testing as assessed by flow cytometric immunophenotyping. Upper panels (a and b) indicate the BM cell bulk before CliniMACS selection and lower panels (c and d) indicate the positive fraction of cells obtained after CliniMACS selection and overnight storage. Briefly population of interest is initially gated by a dual light scatter dot plot (panels a and c); CD34^+^/CD133^+^ double positive cells before and after selection are then identified and calculated by dual fluorescence dot plot analysis as indicated in panels (b) and (d), respectively.

Eight out of 8 batches resulted to be fully compliant according to the predefined acceptance criteria and they were released by a qualified person.  Table 3 reports a summary of the release data obtained in all manufactured ATMP-CD133 batches. A representative example of CD34^+^/CD133^+^ cells characterization is illustrated in Figure 2, representing a simplified gating strategy adopted to determine purity of CD34^+^/CD133^+^ cells.

### 3.4. Drug Substance Stability

CD133^+^ cells viability after overnight storage in X-VIVO15 has been systematically tested on each batch as shown in Table 4. An overnight incubation has been scheduled as necessary because cell production and quality test required several hours and they generally can be completed not before 7.00 pm. The surgical procedure was then scheduled the day after BM harvesting and processing. The overnight storage in X-VIVO has been validated for maintenance of cell viability without addition of any cytokines. This procedure allowed us to maintain the composition of the ATMP-CD133 untouched, while limiting the risk of zoonosis and/or adventitious virus contamination related with the use of FBS. Viability was found in compliance with the acceptance criteria of ≥70% for all tested batches. These data allow to confirm that drug substance, resuspended in X-VIVO15 and storage overnight at +2/+8°C, is stable for at least 14 h, which represents the time lapse before resuspending cells in physiological saline + 5% HSA. A summary of drug substance stability results is reported in Table 4.

### 3.5. Medicinal Product Stability

Stability was assessed retrospectively in 3 out of 8 batches. To this purpose, we thawed ATMP-CD133 frozen samples stored in liquid nitrogen at −196°C. After thawing CD133^+^ cells were resuspended in physiological saline + 5% HSA and maintained up to 12 h. As reported in Table 5, the expected cell viability of at least 70% was obtained at any time point with values fulfilling the established threshold (mean 92.41% ± 1.22%). In addition the medicinal product demonstrated maintaining the expected immunophenotype up to 12 h, as shown in Table 5.

### 3.6. Partitioning of ATMP-CD133 Final Product

The final amount of cells shipped for clinical use was determined based on QC tests detailed in Table 6 in which a summary of cell partitioning is reported. The median of total cells after CliniMACS selection and overnight storage in X-VIVO15 was 9.69 × 10^6^ (range 5.16 × 10^6^–40.14 × 10^6^). As described in FPC test (see Supplemental Table 2), a median of 2.40 × 10^6^ cells (range 1.35 × 10^6^–3.73 × 10^6^) was collected to perform QC tests for batch release whereas the median amount of cells frozen for prospective QC testing was 0.96 × 10^6^ (range 0.54 × 10^6^–5.58 × 10^6^). Hence, the final amount of cells delivered to the clinical site was 6.66 × 10^6^ (range, 2.85 × 10^6^–30.84 × 10^6^).

## 4. Discussion

In November 2011, CAT/EMA agencies published a scientific recommendation concerning the classification of autologous BM-MNCs and autologous BM-derived CD133^+^ cells intended for the treatment of postacute myocardial infarction and chronic ischemic heart disease as ATMPs [31]. Specifically, according to these guidelines, ATMP-CD133 comes under the definition of a somatic cell-based medicinal product to be manipulated as a drug. It is worth to note that the development of ATMP needs a consistent validation of cell product safety and potency. Moreover, its employment into a specific clinical setting requires taking into account several pieces of information such as the autologous/allogeneic use, the starting material, and the donors/recipient characteristics. Yet, translation of the ATMP-CD133 in a full-GMP setting needs a complete standardization of manufacturing steps as well as a robust validation of the release criteria. We have previously set preliminary clinical-grade manufacturing conditions to select cord blood derived-CD133^+^ cells for cardiovascular regenerative purposes [26]. We provided a first evidence of manufacturing process reproducibility, setting specification for batches release, and the proof-of-principle of both* in vitro* and* in vivo* potency of ATMP-CD133 [26].

However, for a cardiac autologous clinical application, these data needed to be confirmed in the context of BM starting material of autologous origin, possibly taking into an account the specificity of patients as self-donors. Multiple cardiovascular risk factors, including aging, diabetes, and smoking habit, are in fact well known to severely affect levels and biology of PB and BM progenitors [24, 32]. Consequently, the present work aimed to generate the validation of a full GMP-grade manufacturing process to produce BM-derived CD133^+^ cells for autologous cardiac cell therapy, taking advantage of the BM of patients dealing with refractory ischemic cardiomyopathy as starting material. Given the nature of compassionate therapy of the first-in-man study, no further validation of the ATMP-CD133 potency has unfortunately been allowed by the local Ethical Committee. This experiment will be carried out in the context of ongoing RECARDIO Trial (clinicaltrials.gov, Identifier: NCT02059681).

Based on our preclinical data [26] and the previous pilot clinical experience [33], we believe that the best recipients of ATMP-CD133 are patients with chronic ischemic HF not suitable to conventional treatments showing a significant amount of reversible ischemia in LV territories. In our pilot clinical experience with ATMP-CD133 in no-option angina patients, we were unable to correlate number of cells injected and angina frequency rate, given the low number of patients treated. This issue is however considered as secondary end-point in the ongoing RECARDIO Trial (clinicaltrials.gov, Identifier: NCT02059681).

As for the manufacturing process reproducibility, the high variability in CD133^+^ cell number, related to the high heterogeneity of an elder and sick cardiovascular patient population, is probably the major shortcoming of this therapeutic approach [24, 25, 34]. We have previously shown [14] that cellularity varied among published studies using BM-derived CD133^+^ in a cardiac setting from 1.5 up to 16.9 × 10^6^. In the recent CARDIO133 trial [3], Nasseri et al. have reported a cell infusion range of 3–9.1 × 10^6^, with a mean of 5.1 × 10^6^ cells. The relatively low number of cell injected has been advocated by the authors as a possible cause of a limited therapeutic effect. In our process, we were able to purify a mean of 10.37 × 10^6^ (range 2.85 × 10^6^–30.84 × 10^6^) of BM-derived CD133^+^ cells, thus increasing the number of cells available per patient. A possible partial explanation for this difference may be the more stringent standardization of BM volume collection, thus reducing the high variability among samples described in CARDIO133 trial.

Notably, in-process critical steps are also present throughout the sequential passages of product manipulation in which an amount of cellular loss is strictly related to the adopted purification protocols. Two critical steps (i.e., Ficoll centrifugation and CliniMACS selection) are responsible for a median cellular loss of 35.11% (range 14.79%–61.75%) and 69.95% (range 43.23%–81.63%), respectively. The ATMP-CD133 recovery has then been improved step by step during productions, reaching a cellular loss as low as 35.38% and 43.23% in Ficoll and CliniMACS selection, respectively. It is worth to note that we always achieved a number of cells fitting the threshold for clinical use, regardless of the cell amount for QC testing. As described in Table 6, a median of 3.36 × 10^6^ of ATMP-CD133 cells is needed to perform QC tests. Moreover, considering the minimal manipulation of BM to select CD133^+^ cells and the absence of an extensive expansion of cells, mycoplasma testing will be omitted from standard release criteria in our current protocols (see FPC2 Supplemental Table 2), thus saving 1 × 10^6^ CD133^+^ cells* per* batch injectable to the patient.

As for other relevant batch release criteria, as compared to our previous protocol [26], we have further increased the process efficiency, improving cell purity from 50% to 70% and viability from 70% to 80%. Thus, the final batch formula adopted for ATMP-CD133 in the RECARDIO trial [27] is defined as follows: purity ≥ 70%, viability ≥ 80%, cellularity between 1 and 12 × 10^6^ cells, sterile, and endotoxin-free. For both purity and viability criteria a median value ≥ 90% has been obtained. Our standard quality parameters, purity and viability thresholds, are in agreement with data from previously published clinical trials using CD133^+^ cells in a cardiologic setting, which adopted thresholds of at least 70% and 80% in cell purity and viability, respectively [4, 11, 35, 36]. As for cell dose, according to our first-in-man experience and data available in literature [4, 13, 37], we agreed about safety purposes with the competent Italian regulatory body (Istituto Superiore di Sanità, ISS) to set at 10 × 10^6^ the highest CD133^+^ cells dose. Considering ≥80% as minimal purity threshold obtained in our preparations, the final total ATMP-CD133 cell maximum number has been then established at 12 × 10^6^. As suggested by ISS, the minimal cell dose has been set at 1 × 10^6^ in line with published studies in the same clinical setting [6, 9–12, 38]. Importantly, this cell range has been verified as sustainable in our GMP conditions.

Finally, according to ATMP recommendations, to ensure the highest quality consistency as well as safety of the final medicinal product, we have provided a specific characterization and monitoring of product impurities for each ATMP-CD133 batches produced. In summary, in this work we have optimized the ATMP-CD133 manufacturing process. However, in this protocol, Ficoll separation and CliniMACS selection are the most critical manipulation steps, representing a bottleneck for total cell recovery. Technological improvements are currently under investigation to overcome these limitations.

## 5. Conclusions

In conclusion, this work has been prepared to provide the full-GMP compliant manufacturing validation of bone marrow-derived human CD133^+^ cells to be used as ATMP for cardiac cell therapy. This work may represent a platform for any future study of cell therapy with CD133^+^ progenitors in the cardiologic field.

## Supplementary Material

Table S1. Parameters relative to demographic and clinical characteristic variables of treated patients. Demographic variables include age and sex; clinical characteristics consist of cardiac surgical and percutaneous intervention and concomitant medications.
Table S2. Details of quality control tests performed throughout the manufacturing steps both for drug substance (in-process controls, IPC) and medicinal product (final-product controls, FPC).
Table S3. Upper table summarize characteristics (reactivity, clone number, conjugated fluorochrome, and brand) of monoclonal antibodies used for immunphenotyping; Lower table include combination of antibodies used for immunphenotyping:
- Tube 1 contain only propidium iodide and represent the negative control for CD34, CD133 and CD45 staining.
- Tube 2 contains CD34, CD45, IgG2b antibodies and propidium iodide and represent the minus 1 control strategy with the whole marked staining excepted for the more important antigen, in our case CD133.
- Tube 3 contains CD34, CD45 and CD133 antibodies and propidium iodide for the identification of positive population.
Table S4. Details of release test, methods used and acceptance criteria applied to ATMP-CD133 final product.

## Figures and Tables

**Table 1:** Purity and viability of CD133^+^ cells (%) obtained in 8 batches of ATMP-CD133.

Batch #	% CD133^+^		Cell viability	
	Starting material	Final product (purity)	Starting material	Final product
PTC-CD133-052	1.87	96.2	95.83%	96.03%
PTC-CD133-055	3.39	81.87	96.83%	97.70%
PTC-CD133-068	0.86	94.61	95.27%	99.87%
PTC-CD133-085	1.18	89.80	87.67%	99.91%
PTC-CD133-090	0.41	81.40	96.62%	99.75%
PTC-CD133-098	0.90	91.40	93.62%	99.95%
PTC-CD133-109	1.21	93.64	96.28%	99.97%
PTC-CD133-116	0.90	81.94	96.29%	99.86%

Mean	1.34	88.86	94.80%	99.13%
Standard deviation	0.93	6.21	3.06%	1.47%
Median	1.04	90.60	96.06%	99.87%
Minimum value	0.41	81.40	87.67%	96.03%
Maximum value	3.39	96.2	96.83%	99.97%

**Table 2:** Recovery of CD133^+^ cells.

Batch #	Starting material	Pre-CliniMACS selection^*∗*^	Post-CliniMACS selection	
	Absolute number of CD133^+^ cells (×10^6^)	Cell recovery 1 (%)^*∗∗*^	Cell recovery 2 (%)^#^	Cell recovery 3 (%)^§^
PTC-CD133-052	180.69	50.29	18.37	9.24
PTC-CD133-055	123.68	38.25	20.10	7.69
PTC-CD133-068	38.13	85.21	30.05	25.61
PTC-CD133-085	44.89	78.99	20.69	16.34
PTC-CD133-090	15.40	70.22	35.45	24.89
PTC-CD133-098	65.81	51.23	30.05	15.40
PTC-CD133-109	97.54	68.17	51.20	34.90
PTC-CD133-116	40.61	61.62	56.77	34.98

Mean	75.84	63.00	32.83	21.13
Standard deviation	54.94	15.78	14.40	10.65
Median	55.35	64.89	30.05	20.62
Minimum value	15.40	38.25	18.37	7.69
Maximum value	180.69	85.21	56.77	34.98

^*∗*^Preselection is referred to cells obtained after Ficoll centrifugation.

^*∗∗*^Cell recovery from starting material.

^#^Cell recovery from pre-CliniMacs selection.

^§^Cell recovery from starting material.

**Table 3:** Summary of release data in 8 ATMP-CD133 batches.

Release test	Results				Acceptance criteria
	Mean	Standard deviation	Median	Range	
Purity (%)	88.86	±6.21	90.60	81.40–96.20	≥50%
Viability (%)	99.13	±1.47	99.87	96.03–99.97	≥70%
Cellularity (×10^6^)	10.37	±9.08	9.69	2.85–30.84	≥1.0 × 10^6^
Sterility	Sterile (8/8 batches)				Sterile
Endotoxin	<0.5 EU/mL (8/8 batches)				<0.5 EU/mL
Mycoplasma	Absent (8/8 batches)				Absent

**Table 4:** Stability of ATMP-CD133 during overnight storage in X-VIVO15.

Batch #	Time of overnight storage	Cell viability
	(hours:minutes)	(% of live cells)
PTC-CD133-052	12:15	96.03
PTC-CD133-055	13:55	97.70
PTC-CD133-068	12:43	99.87
PTC-CD133-085	13:37	99.91
PTC-CD133-090	12:35	99.75
PTC-CD133-098	13:05	99.95
PTC-CD133-109	13:15	99.97
PTC-CD133-116	13:49	99.86

**Table 5:** Stability of CD133^+^ cells in physiological saline (+5% HSA) at 4°C in three ATMP-CD133 batches.

Batch #	Parameter	Time points			
		*T*0 (thawing)	*T* + 6 h	*T* + 9 h	*T* + 12 h
PTC-CD133-098	Viability (%)	97.00	91.68	90.76	91.07
	Purity (% CD133^+^ cells)	92.76	nd	92.73	90.59

PTC-CD133-116	Viability (%)	90.72	94.23	94.07	93.47
	Purity (% CD133^+^ cells)	84.69	nd	85.98	85.86

PTC-CD133-068	Viability	88.70	90.00	94.70	92.68
	Purity (% CD133^+^ cells)	89.15	nd	89.41	87.89

nd: not done.

**Table 6:** Summary cell recovery according to processing phase.

Batch #	Number of total cells^*∗*^ after selection (×10^6^)	Number of cells sampled for all QC test (×10^6^)	Number of frozen cells for QC retesting (×10^6^)	Number of cells shipped for clinical use (×10^6^)
PTC-CD133-052	20.63	3.55	4.5	12.58
PTC-CD133-055	8.16	2.18	0.54	5.44
PTC-CD133-068	7.98	1.35	0.79	5.84
PTC-CD133-085	6.92	1.84	0.58	4.5
PTC-CD133-090	5.16	1.62	0.69	2.85
PTC-CD133-098	11.22	2.62	1.12	7.48
PTC-CD133-109	40.14	3.73	5.58	30.84
PTC-CD133-116	17.82	2.77	1.62	13.43

Mean	14.75	2.46	1.93	10.37
Standard Deviation	11.61	0.87	1.97	9.08
Median	9.69	2.40	0.96	6.66
Minimum Value	5.16	1.35	0.54	2.85
Maximum Value	40.14	3.73	5.58	30.84

^*∗*^Number of cells obtained after CliniMACS selection and overnight storage in X-Vivo15.
