# Supplementary material for: Full GMP-Compliant Validation of Bone Marrow-Derived Human CD133+ Cells as Advanced Therapy Medicinal Product for Refractory Ischemic Cardiomyopathy
Source: Biomed Res Int. 2015 Oct 1;2015:473159. doi: 10.1155/2015/473159 (PMC4606188; doi:10.1155/2015/473159)
Supplement: Supplementary file 1 — Table S1. Parameters relative to demographic and clinical characteristic variables of treated patients. Demographic variables include age and sex; clinical characteristics consist of cardiac surgical and percutaneous intervention and concomitant medications. Table S2. Details of quality control tests performed throughout the manufacturing steps both for drug substance (in-process controls, IPC) and medicinal product (final-product controls, FPC). Table S3. Upper table summarize characteristics (reactivity, clone number, conjugated fluorochrome, and brand) of monoclonal antibodies used for immunphenotyping; Lower table include combination of antibodies used for immunphenotyping: - Tube 1 contain only propidium iodide and represent the negative control for CD34, CD133 and CD45 staining. - Tube 2 contains CD34, CD45, IgG2b antibodies and propidium iodide and represent the minus 1 control strategy with the whole marked staining excepted for the more important antigen, in our case CD133. - Tube 3 contains CD34, CD45 and CD133 antibodies and propidium iodide for the identification of positive population. Table S4. Details of release test, methods used and acceptance criteria applied to ATMP-CD133 final product. [file 473159.f1.doc]

SUPPLEMENTAL TABLE 1: Patient characteristics

| **Number of patients** | 8 |
| --- | --- |
| **Age (years)** | 63.5 ± 8 (48 – 72) |
| **Men** | 7/8 (87.5%) |
| **PCI** | 5/8 (62.5%) |
| **CABG** | 6/8 (75%) |
| **SCS** | 2/8 (25%) |
| **Risk factors** |  |
| **Hypertension**  **Diabetes**  **Dyslipidemia**  **History of smoking**  **Positive family history** | 8/8 (100%)  2/8 (25%)  6/8 (75%)  4/8 (50%)  6/8 (75%) |
| **Medications** |  |
| **Beta-blockers**  **Angiotensin-converting enzyme inhibitor**  **Calcium channel blockers**  **Statins** | 8/8 (100%)  2/8 (25%)  4/8 (50%)  6/8 (75%) |

PCI: percutaneous coronary angioplasty, CABG: coronary artery bypass, SCS: Spinal Cord Stimulator.

SUPPLEMENTAL TABLE 2: Summary of in-process and final controls.

| **Sample** | **Step** | **Test** | **Method** | **Equipment** |
| --- | --- | --- | --- | --- |
| IPC1 | Starting material | Cell count | Automatic counting | ACT-diff, Beckman Coulter |
|  |  | Viability and immunophenotype | Flow Cytometry | FACS Calibur, Becton Dickinson |
| IPC2 | Waste from bone marrow washing | Cell count | Automatic counting | ACT-diff, Beckman Coulter |
| IPC3 | Waste from MNCs washing | Cell count | Automatic counting | ACT-diff, Beckman Coulter |
| IPC3.1 | Waste from MNCs washing (only if IPC3 > 1x10^3^/µl) | Cell count | Automatic counting | ACT-diff, Beckman Coulter |
| IPC4 | Waste from MNCs washing | Cell count | Automatic counting | ACT-diff, Beckman Coulter |
| IPC4.1 | Waste from MNCs wash (only if IPC4 > 1x10^3^/µl) | Cell count | Automatic counting | ACT-diff, Beckman Coulter |
| IPC5 | Waste from wash post-antibody incubation | Cell count | Automatic counting | ACT-diff, Beckman Coulter |
| IPC5.1 | Waste from washed post-antibody incubation (only if IPC5 > 1x10^3^/µl) | Cell count | Automatic counting | ACT-diff, Beckman Coulter |
| IPC6 | Pre-selection fraction | Cell count | Automatic counting | ACT-diff, Beckman Coulter |
|  |  | immunophenotype | Flow Cytometry | FACS Calibur, Becton Dickinson |
| IPC7 | Post-selection negative fraction | Cell count | Automatic counting | ACT-diff, Beckman Coulter |
|  |  | immunophenotype | Flow Cytometry | FACS Calibur, Becton Dickinson |
| IPC8 | Waste of positive-fraction centrifugation | Cell count | Automatic counting | ACT-diff, Beckman Coulter |
| IPC8.1 | Waste of positive-fraction centrifugation (only if IPC8 > 1x10^3^/µl) | Cell count | Automatic counting | ACT-diff, Beckman Coulter |
| IPC9 | Positive-fraction | Cell count, | Trypan Blue dyes exclusion | Zeiss |
|  |  | Viability and immunophenotype | Flow Cytometry | FACS Calibur, Becton Dickinson |
| FPC1 | Overnight positive fraction | Cell count, | Trypan Blue dyes exclusion | Axioscope 40, Zeiss |
|  |  | Viability and immunophenotype | Flow Cytometry | FACS Calibur, Becton Dickinson |
| FPC2 | Overnight positive fraction | Mycoplasma | Culture Method (EU Ph 2.6.7) | - |
| FPC3 | Overnight positive fraction | Endotoxin | Gel clot LAL assay (EU Ph 2.6.14) | - |
| FPC4 | Overnight positive fraction | Sterility | Microbiological control of cellular products ( EU Ph 2.6.1) | - |

IPC: In Process Control, FPC: Final Product Control

SUPPLEMENTAL TABLE 3: Characteristics and combination of monoclonal antibodies used for immunphenotyping

| **Selected Antibodies** | | | | | | | |
| --- | --- | --- | --- | --- | --- | --- | --- |
| **Reactivity** | | **Clone** | | **Fluorochrome** | **Source** | | |
| FcR Blocking | | - | | - | Miltenyi Biotec | | |
| CD45 | | 5B1 | | FITC | Miltenyi Biotec | | |
| IgG2b | | 27-35 | | PE | Becton Dickinson | | |
| CD133 | | 293C3 | | PE | Miltenyi Biotec | | |
| CD34 | | AC136 | | APC | Miltenyi Biotec | | |
| **Staining Panels Immunophenotype** | | | | | | | |
| Tube 1 | FcR Blocking | | - | | - | Propidium Iodide | - |
| Tube 2 | FcR Blocking | | CD45 FITC | | IgG2b PE | Propidium Iodide | CD34 APC |
| Tube 3 | FcR Blocking | | CD45 FITC | | CD133 PE | Propidium Iodide | CD34 APC |

FcR : Fc receptor

SUPPLEMENTAL TABLE 4: Release test and acceptance criteria applied to ATMP-CD133 final product

| **Test** | **Method** | **Acceptance criteria** |
| --- | --- | --- |
| Purity | Flow Cytometry | ≥50% |
| Viability | Flow Cytometry | ≥70% |
| Cellularity  (number of total viable nucleated cells) | Trypan blue dye exclusion | ≥1.0 x 10^6^ |
| Endotoxin | Gel Clot LAL Assay  (EU Pharmacopeia 2.6.14) | < 0.5 EU/mL |
| Sterility | Sterility  (EU Pharmacopeia 2.6.1) | Sterile |
| Mycoplasma | Mycoplasma, culture method  (EU Pharmacopeia 2.6.7) | Absent |
